# Evaluation of Monitoring Traps and Lures for *Drosophila suzukii* (Diptera: Drosophilidae) in Berry Plantings in Florida

**DOI:** 10.3390/insects10100313

**Published:** 2019-09-24

**Authors:** Dasia S. Harmon, Muhammad Haseeb, Lambert H. B. Kanga, Oscar E. Liburd

**Affiliations:** 1 FoodCorps, Inc., Atlanta, GA 30303, USA; harmondasia@gmail.com; 2 Center for Biological Control, College of Agriculture and Food Sciences, Florida A&M University, Tallahassee, FL 32307, USA; lambert.kanga@famu.edu; 3 Entomology and Nematology Department, University of Florida, Gainesville, FL 32611, USA; oeliburd@ufl.edu

**Keywords:** spotted-wing drosophila, detection, trapping, pest management, blueberry, blackberry

## Abstract

*Drosophila suzukii* (Diptera: Drosophilidae) is an invasive insect pest that was detected in Florida in August 2009 in Hillsborough County. Very limited information is available for berry growers to properly detect and monitor this serious pest in southern highbush blueberry (hybrids of *Vaccinium corymbosum* L. × *V. darrowi* Camp), rabbiteye blueberry (*Vaccinium virgatum* L.), and blackberry (*Rubus fruticosus* L.) production systems. We compared several *D. suzukii* traps and lures/baits at two sites in Florida. The traps evaluated included Trécé, Scentry, and a standard homemade cup trap. These traps were compared with various baits and lures, including Trécé lure, Scentry lure, yeast bait, and Suzukii trap, under Florida production systems. Early detection is important to develop an effective monitoring system so management action can be taken before economic damage occurs. Data were recorded as overall trends, as well as in 4–5 trapping periods from early to late season. Overall, the Scentry trap baited with Scentry lure, the Trécé trap baited with Trécé lure + yeast, and the Trécé trap baited with Scentry lure were the best performing traps. Yeast-based traps were also attractive to *D. suzukii* early in the season, but they did not provide consistent captures as the season progressed. The Scentry trap with yeast bait, the Scentry trap with Scentry lure, the Trécé trap with Trécé lure + yeast bait, and a cup trap with yeast bait caught most of the flies during the first trapping period in 2015 and 2016 in the rabbiteye blueberry. In the southern highbush blueberry, the population of *D*. *suzukii* was much lower than in the rabbiteye blueberry planting, and the Scentry trap with Scentry lure captured the highest number of flies during the first trapping period in 2016. In the blackberry, the Scentry trap with Scentry lure numerically had the highest captures during the first trapping period, but this was not significantly different from the cup trap with yeast bait, the Trécé trap baited with Suzukii trap, and the Trécé trap with Trécé lure. Overall, the Scentry trap with Scentry lure was the most consistent trap that captured *D. suzukii* flies throughout the season in the three production systems—rabbiteye blueberry, southern highbush blueberry, and blackberry. Growers in low pressure systems that are similar to Florida can use the Scentry trap with Scentry lure to monitor *D. suzukii* populations.

## 1. Introduction

The production of berry fruits, especially blueberries and blackberries, is increasing in Florida and other southern States in the United States. In 2017, 33,953 hectares of blueberries with a value of $720 million were cultivated in the United States. During the same year, Florida’s growers produced blueberries and blackberries on more than 1821 hectares with a value of $53.7 million (USD) [1]. The demand for growing these berry fruits is partially due to their health benefits and profits to producers, and as an alternative to citrus production, which has been severely damaged by citrus greening (Huanglongbing) disease.

The spotted-wing drosophila, *Drosophila suzukii* (Matsumura) (Diptera: Drosophilidae), is considered to be one of the most serious, invasive insect pests of thin-skinned fruits. *Drosophila suzukii* was first recorded in Hillsborough County, Florida, in 2009 [2]. The species is native to Southeast Asia and is now widely distributed in Asia, North America, South America, and Europe [3,4,5]. Unlike other drosophilid flies, the ovipositor of the *D. suzukii* female is enlarged and serrated, allowing it to cut into thin-skinned fruits and lay eggs. Kanzawa [6] observed that the fly oviposited most often on cherries, peaches, plums, persimmons, strawberries, and grapes in Japan but was also opportunistic and would feed on fallen fruits on the ground that were spoiled or fermented [7]. In the United States, *D. suzukii* has now been documented on more than 25 host plants, including blueberries and caneberries (raspberry and blackberry), the latter being among the most susceptible hosts [8]. While it is difficult to accurately estimate the economic damage due to *D. suzukii* in the United States, it has been reported that the damage may be $850–900 million annually [9]. An effective and efficient trap-and-lure system is critical to detect adult flies in the field before they begin to lay eggs and to reduce economic losses. The detection of one larva in a shipment of berries can result in the total rejection of the product in the market and a significant loss of income and trade.

Several trap-and-lure systems have been evaluated for monitoring *D. suzukii*. Lee et al. [10] compared various trap designs in seven US states and found that fly captures among trap designs were different per site. A Haviland trap caught the highest number of *D. suzukii* flies followed by a red Van Steenwyk, and a clear trap. Traps with larger entry holes caught more flies than traps with smaller entry areas [10,11,12]. Iglesias et al. [13] evaluated the attractiveness of several trap designs, bait types, and bait age in southern highbush and blackberries and found that a cup trap baited with yeast + sugar captured the most *D. suzukii* over other vinegar-based baits. Further research showed a significant drop in the capture rates of drosophilid flies when the traps were left in the field for more than one week.

Fermented odors of wine, yeast, vinegar, and sugar have been used extensively as baits for monitoring *D. suzukii* flies [13,14]. Burrack et al. [14] compared a range of homemade lures—a fermenting cup consisting of yeast, whole wheat flour and apple cider vinegar (ACV) hung over a drowning solution, and a synthetic lure (Trécé + ACV)—across 10 states and found that the fermenting bait cup and synthetic lure captured most of the flies (pooled male and female data) across all states and host plantings. All of the attractants evaluated captured flies at least one week earlier than apple cider vinegar.

With respect to wine-based lures, Landolt et al. [15] baited traps with wine and vinegar in separate treatments, as well as in treatments where wine and vinegar were mixed together. They found that traps baited with mixtures of wine and vinegar caught significantly more *D. suzukii* flies than traps baited with either compound alone. Mixtures of wine and vinegar also performed better than a mixture of acetic acid and ethanol in water treatment. Using field and laboratory experiments, Cha et al. [16] identified the volatiles in wine and vinegar that were most attractive to *D. suzukii*. Recently, Jaffe et al. [17] evaluated several types of attractants for monitoring *D. suzukii* and found that yeast- and sugar-based volatiles captured the most *D. suzukii* flies. The addition of a commercial lure to yeast- and sugar-based baits increased the trap captures.

Most of the trap monitoring studies in the United States for *D. suzukii* have been done under high pressure systems, using northern highbush blueberry (*Vaccinium corymbosum* L.). Florida has a low pressure *D. suzukii* system, in which the average fly pressure rarely exceeds 20 flies per trap per week. This is unlike other states with high pressure systems, including North Carolina, New Jersey, and Michigan, in which the average *D. suzukii* fly pressure is >75 flies per trap per week. To our knowledge, no pest monitoring studies with *D. suzukii* have been reported using rabbiteye blueberry (*Vaccinium virgatum* L.) and only one paper [13] with southern highbush blueberry (*V. corymbosum* L. × *V. darrowi* Camp). Both southern highbush and rabbiteye are considered low pressure systems for *D. suzukii* in Florida. The purpose of this study was to evaluate the effectiveness of traps and lures in low pressure rabbiteye and southern highbush blueberry in open berry fields in Florida. Evaluation studies were also conducted in a blackberry planting.

## 2. Materials and Methods

### 2.1. Study Sites

**Figure 1:**
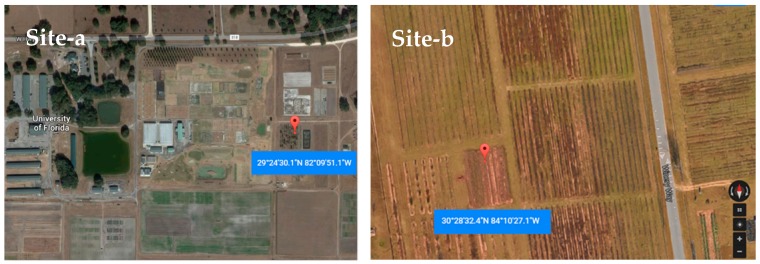
Site-a—monitoring site for *Drosophila suzukii* at the Plant Science Research and Education Unit, University of Florida, Citra, FL. Site-b—the Center for Viticulture and Small Fruits Research, FAMU, Tallahassee, Florida. Photo source: Google maps.

In 2015 and 2016, a rabbiteye blueberry and a blackberry planting were investigated in Citra, Florida. In 2016, a southern highbush blueberry planting was investigated in Tallahassee, Florida (Figure 1, Site-a and Site-b). The southern highbush plants were 4 years old, and the bushes were ~0.9 m tall. The blackberry plants were 7 years old, and the bushes were 1.4 m tall.

### 2.2. Experimental Design

The randomized complete block design was used for the experiments with 4 replicates at each site. All trap treatments were blocked according to the variety of blueberry and blackberry. Therefore, all treatments were exposed to the same varieties to prevent any varietal effects. All traps were deployed in the canopy of the growing blueberry and blackberry bushes. The traps were placed in the field when 5% of the berries showed signs of turning blue. All traps in each experiment were serviced once per week. This included rotating the trap positions in the field to prevent any positional bias within each block. Servicing the traps also included replacing the fly drowning solution with respective treatments and using Tupperware (Tupperware Corporation, Orange Blossom Trail, FL, USA) to collect the solution from the traps. The traps were then taken back to the laboratory to separate the adults of *D*. *suzukii* from the other insect species and to sex the flies. For the experiments that were carried out in Citra during 2015 (blueberry and blackberry), the different trap contents were assessed for comparing males and females relative to the overall mean capture totals that are reported in the figures.

### 2.3. Traps and Lures

**Figure 2:**
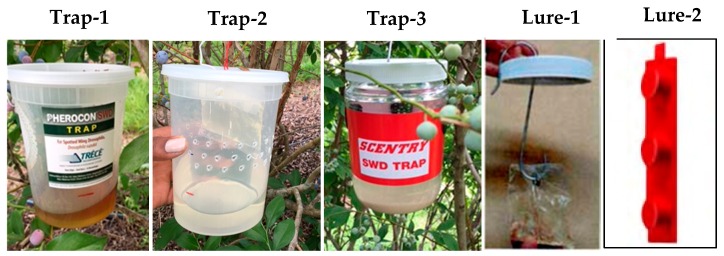
Photos of traps (color and shapes) and lures used in the study: Trap-1 (Trécé trap), Trap-2 (Cup trap), Trap-3 (Scentry trap), Lure-1 (Scentry trap lure), and Lure-2 (Trécé trap lure).

The Trécé trap (Great Lakes IPM, Vestaburg, MI, USA) is marketed as a monitoring device for *D. suzukii*. The Trécé trap is a 0.95 L clear container that comes with a lid and wire hanger (Figure 2, Trap-1). Fly entry points are on each side of the trap. The cup trap was homemade. It was a 0.95 L polypropylene plastic container with a lid made by Delipro (New York, NY, USA) (Figure 2, Trap-2). The cup trap had 3 rows of 17 holes (each hole was 3 mm in diameter) burned into the side of the cup to serve as entry points for the flies. The Scentry trap (Scentry Biological Inc, Billings, MT, USA) is marketed as a monitoring device for *D. suzukii* (Figure 2, Trap-3). The Scentry trap is mostly red and has a white lid and a wire hanger for the lure. Entry holes are on both sides of the jar.

The commercial lures (Trécé and Scentry) used in the study are marketed to last 6 to 8 weeks for the best results. Neither the blueberry nor blackberry field experiments extended beyond this time so there was no need to replace the lures during the trap servicing. The manufacturers of both of these products do not provide the chemical contents embedded in the lures and describe them as “proprietary”. The lures contain several components, including acetic acid, ethanol, acetoin, and mathionol (information obtained directly from company representatives). The lure used in our study known as “Suzukii trap” was not commercially available during the time of the study and was only used for research purposes. This product is composed of 7% hydrolyzed protein and 2% organic acid. The remaining ingredients are proprietary and assigned to the manufacturers by law. The yeast bait was brewer’s yeast (*Saccharomyces cerevisiae*)—a one-celled fungus. The detergent solution was 98% water with 2% odorless detergent (Publix Super Market, Gainesville, FL, USA). The yeast bait and the detergent solution were replaced weekly. The combination of different types of traps and lures were tested to determine their efficiencies in trapping *D. suzukii* adults in berry plantings. The details of the various treatments used in the study are provided in Table 1.

### 2.4. Effectiveness of Nine Trapping Systems to Detect D. suzukii in Rabbiteye Blueberry in Citra, Florida

The experiment was conducted in rabbiteye blueberries (*Vaccinium virgatum*) during the 2015 and 2016 fruiting season from May to June. The blueberry bushes were approximately 8 years old. During this study, the blueberry plants were approximately 2 m in height and 1 m apart with 1.5 m between rows that were approximately 50 m long. The experimental blocks were approximately 5 m apart. The treatments were blocked by rabbiteye cultivars in a randomized block design. Four rabbiteye cultivars were included—“Climax”, “Brightwell”, “Premier”, and “Powderblue”. In this experiment, we determined the effectiveness of nine trapping systems for monitoring *D*. *suzukii* (Table 1).

### 2.5. Effectiveness of Seven Trapping Systems to Detect D. suzukii in Southern Highbush Blueberry (SHB) in Tallahassee, Florida

The experiment was conducted in southern highbush blueberries (*Vaccinium corymbosum* L. × *V. darrowi* Camp) during the 2016 fruiting season (April–May) in Tallahassee, Florida, at the Florida A&M University’s Center for Viticulture and Small Fruits Research Center. The blueberry bushes in Tallahassee were four years old. The experimental plots of blueberry bushes comprised 8 rows. Each row consisted of 24 southern highbush blueberry plants. The experimental blocks were approximately 5 m apart, and the blueberry rows were approximately 3 m apart. Each row was approximately 100 m long, and the bushes were spaced 1.5 m apart. On average, the plants were 1.5 m tall. The cultivars used in this experiment consisted of “Meadowlark”, “Star”, “Emerald”, “Farthing”, “Sweetcrisp”, and “Abundance”. The data for Experiment 2 were collected over the course of 7 weeks. In this experiment, we determined the effectiveness of 7 trapping systems for monitoring *D*. *suzukii* (Table 1).

### 2.6. Effectiveness of Six Trapping Systems to Detect D. suzukii in Blackberry Planting in Citra

This experiment was conducted during May to June in Citra, Florida, in blackberries. The rows of blackberries were approximately 100 m long. The plants were on a trellis system and were approximately 2 m in height. These bushes were approximately 7 years old. The traps were installed within the bushes away from direct sunlight. In Florida, blackberries typically ripen during May and June. The blackberry varieties used in this study included “Arapaho”, “Chickasaw”, “Kiowa”, and “Ouachita”. The data were collected over the course of 4 weeks during the blackberry growing season. In this experiment, we determined the effectiveness of 6 trapping systems for monitoring *D*. *suzukii* (Table 1).

### 2.7. Data Analysis

The data from each experiment were analyzed separately using a two-way repeated measure analysis of variance (ANOVA) [17]. The counts of *D. suzukii* were transformed to *ln* (x + 0.5) to normalize the distribution and homogenize the variances before analysis. The male and female data were separated during the counts and analyzed separately to determine the effects of the treatments on each sex of *D. suzukii*. The treatment means were separated by Tukey’s HSD (honestly significant difference) test [18].

## 3. Results

### 3.1. Effectiveness of Nine Trapping Systems to Detect D. suzukii in Rabbiteye Blueberry Planting

**Figure 3:**
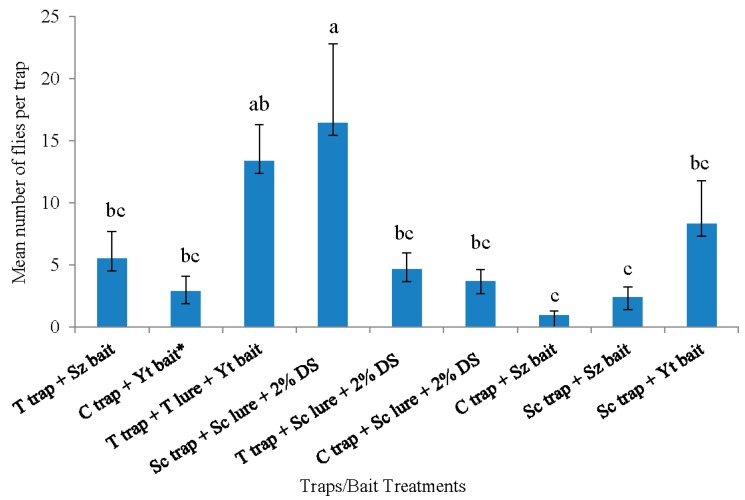
Pooled data of *Drosophila suzukii* adults captured (both sexes) with different trapping systems from all trapping periods in rabbiteye blueberry bushes from 26 May 2015 to 30 June 2015 in Citra, Florida, USA. Mean (± SE) number of adult flies with different letters are significantly different (*p* ≤ 0.05; Tukey’s HSD (honestly significant difference) test from each other. T trap = Trécé trap, T lure = Trécé lure, Sz bait = Suzukii trap, C trap = Cup trap, Yt bait = Yeast bait, Sc trap = Scentry trap, Sc = Scentry lure, and 2% DS = 2% detergent solution. * Reference treatment.

In 2015, the Scentry traps with Scentry lures and the Trécé traps with Trécé lures + yeast bait captured the highest overall mean number of flies. These two trap-and-lure systems did not differ significantly from each other (Figure 3). The Trécé trap with Trécé lure + yeast treatment was not different from any other trapping systems, except the cup trap with Suzukii trap and the Scentry trap with Suzukii trap, which did not differ significantly from one another (Figure 3). 

During 2015, with the exception of the Trécé trap with Trécé lure + yeast bait, the Scentry trap with Scentry lure captured significantly more males compared with the other treatments (F = 4.67; df = 44, 135; *p* = 0.0001). The Trécé trap with Trécé lure and yeast bait captured significantly more females than all other treatments, except the Scentry trap and Scentry lure (F = 3.89; df = 44, 135; *p* = 0.0001) (Table 2). The lowest number of flies (male and female) was recorded on the cup trap with Suzukii trap, the Scentry trap with Suzukii trap, and the cup trap with Scentry lure, which did not differ significantly from one another (Table 2).

**Figure 4:**
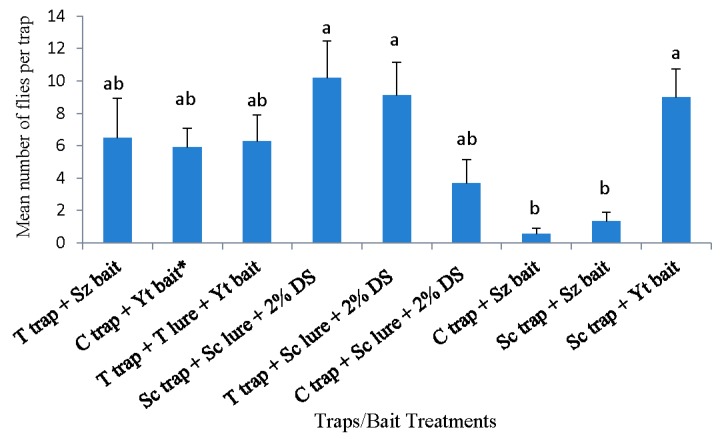
*Drosophila suzukii* flies captured in blueberry planting from 19 May 2016 to 23 June 2016 in Citra, Florida. Mean (± SE) number of adult flies within columns with different letters are significantly different (*p* ≤ 0.05; Tukey’s HSD test) from each other. T trap = Trécé trap, T lure = Trécé lure, Sz bait = Suzukii trap, C trap = Cup trap, Yt bait = Yeast bait, Sc trap = Scentry trap, Sc = Scentry lure, and 2% DS = 2% detergent solution. * Reference treatment.

For the Citra blueberries in 2016, the Scentry trap with Scentry lure, the Trécé trap with Scentry lure, and the Scentry trap with yeast bait captured significantly more flies than the cup trap with Suzukii trap and the Scentry trap with yeast bait (F = 6.2; df = 8, 27; *p* = 0.03). None of the other treatments were significantly (*p* > 0.05) different from each other (Figure 4).

With the exception of the Trécé trap with Trécé lure + yeast bait and the Trécé trap with Scentry lure, more male *D. suzukii* were captured on the Scentry trap with Scentry lure than any other trapping systems evaluated (Table 3). With regards to female *D. suzukii*, the cup trap with yeast bait captured more flies than the cup trap with Suzukii trap, the cup trap with Scentry lure, and the Scentry trap with Suzukii trap (Table 3).

### 3.2. Effectiveness of Seven Trapping Systems to Detect D. suzukii in Southern Highbush Blueberry (SHB) in Tallahassee, Florida

**Figure 5:**
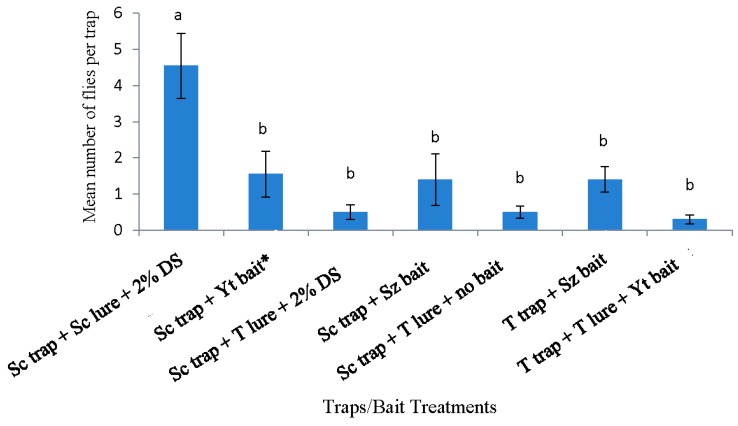
*Drosophila suzukii* adults captured in blueberry planting from 18 April 2016 to 23 May 2016 in Tallahassee, Florida. Yt = Yeast bait, T lure = Trécé lure, Sz = Suzukii trap, Sc lure = Scentry lure, and 2% DS = 2% detergent solution. * Reference treatment.

The overall pooled data (18 April to 23 May) collected in southern highbush blueberry in 2016 Tallahassee, Florida, indicate that the Scentry trap with Scentry lure captured significantly more flies than all other trapping systems (F = 5.37; df 2, 21; *p* = 0.0017) (Figure 5). None of the other trapping systems were significantly different (*p* > 0.05) (Figure 5).

There were significantly more male and female flies captured in the Scentry trap with Scentry lure (F = 3.89; df = 34, 105; *p* = 0.0001) when compared with the other six treatments. There were no significant differences in male captures for all treatments except the Scentry trap with Scentry lure (Table 4). The Scentry trap with Scentry lure captured more female *D. suzukii* compared with other treatments (F = 5.19; df = 34, 105; *p* = 0.0001). Significantly more female *D. suzukii* were captured in the Scentry trap + yeast bait when compared to all treatments (Table 4).

### 3.3. Efficiency of Six Trapping Systems in Blackberry Planting in Citra

**Figure 6:**
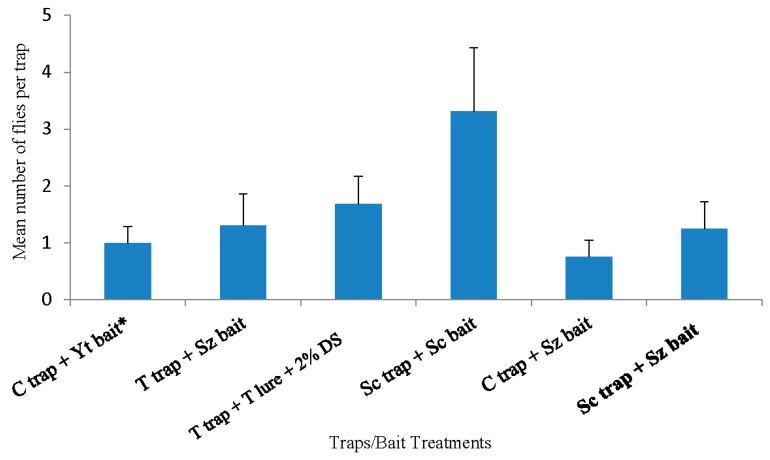
Spotted-wing drosophila flies captured in blackberry planting from 4 June 2015 to 2 July 2015 in Citra, Florida. The letters above the bar indicate significant differences (*p* ≤ 0.05). C trap = Cup trap, Yt = Yeast bait, T trap = Trécé trap, T lure = Trécé lure, Sc trap = Scentry trap, Sc bait = Scentry lure, Sz bait = Suzukii trap, and 2% DS = 2% detergent solution. * Reference treatment.

The Scentry trap with Scentry bait captured significantly more flies than the cup trap with Suzukii trap and the Scentry trap with Suzukii trap (F = 3.20; df = 5, 18; *p* = 0.0307). However, there were no significant differences between the cup trap with yeast bait, the Trécé trap with Suzukii trap, and the Trécé trap with Trécé lure (Figure 6). The cup trap with Suzukii trap and the Scentry trap with Suzukii trap captured the fewest *D. suzukii*, and there was no significant difference between these two treatments. The Scentry trap with Scentry bait captured significantly more flies than other treatments (F = 1.06; df = 5, 18; *p* = 0.0412). The cup trap/yeast trap, Trécé trap/Suzukii bait, Trécé trap/ Trécé lure, and the cup trap/Suzukii bait captured the lowest numbers of male flies. The Scentry trap/Scentry bait treatment captured the most female *D. suzukii* compared to all other treatments (F = 2.28; df = 23, 72; *p* = 0.0043) (Table 5).

## 4. Discussion

Identifying effective monitoring tools is important when developing management programs for invasive pests. Overall, our findings indicate that the Scentry trap baited with Scentry lure, the Trécé trap baited with Trécé lure + yeast, and the Trécé trap baited with Scentry lure were the best performing traps during the 2015 and 2016 studies conducted in rabbiteye blueberries. Traps baited with yeast also performed well initially, and the captures were similar to the Scentry and Trécé traps during the first trapping period in 2015 and the first and second trapping periods in 2016. The problem with the yeast was that the *D. suzukii* captures fell off quickly by the end of the second trapping period during the typical production season. The changes in the attraction to yeast may be related to the reproductive status of the flies. Flies caught early in the season (during the first or second trapping periods) may not be reproductively developed and so may be more attracted to food-based baits, such as yeast [19]. Later in the season (from the third to the fifth trapping period) there were a lot more berries in the field and an abundance of fruit volatiles that ovipositing *D. suzukii* flies may have been attracted to as opposed to yeast.

The attractiveness of yeast early in the season has been previously reported [13,17]. In a recent study, yeast species, strain, and growth media were found to influence the attraction of *D. suzukii* flies to baited traps [20]. Yeast-based baits tend to attract many nontarget insects, including beneficials [13]. These differences in selectivity were quite noticeable when sorting through the samples, but this was not documented. Overall, the authors found that yeast-based baits were often cloudy and had sediments that made fly identification extremely difficult.

The Trécé trap with Trécé lure + yeast was also attractive, but growers would have to purchase all three components (trap, lure, and yeast), and this could become a cumbersome and time-consuming process to combine all three components in the field before deploying the traps.

The Scentry trap and Scentry lure system provided the most consistent captures of *D*. *suzukii* flies in all three production systems (rabbiteye, southern highbush, and blackberry). This trap-and-lure system could be particularly important for monitoring flies, especially in low pressure systems, such as Florida, where fly populations rarely exceed 20 flies per trap.

In our studies in southern highbush blueberry in which the highest *D. suzukii* population was 3.0 ± 1.1 and 4.0 ± 0.7 in the first and second trapping period, respectively, the Scentry trap and Scentry lure captured significantly more flies than all the other trap-and-lure systems evaluated, including the Trécé trap with Trécé lure and an additional yeast bait booster.

The attractiveness of the Scentry trap and Scentry lure may be related to both trap design and lure. The trap is red in color (Figure 2, Trap-3) with several entry holes. During trap evaluation studies, red color traps or darker colored traps performed better than clear traps [21,22,23]. In addition, lure and entry holes were more important in determining attraction than trap color [11,12]. Scentry lure is potentially a mixture of fermented wine vinegar, ethanol, and acetic acid; this information is proprietary for Scentry Biologicals Inc. as indicated by the company representative.

The homemade cup trap did not catch as many flies as the Scentry and Trécé traps in both years, but when baited with Scentry lure or yeast, the cup captures were not significantly different to either of these traps. The performance of the homemade cup trap could be related to the structure and transparency of the trap. The holes may have been insufficient entry pathways to capture enough *D*. *suzukii* flies. Redesigning the trap by making more suitable fly entry holes and changing the color of the trap may improve its performance.

All the traps required preparation work before deploying them in the field. The ease of implementation of the trap varied among the different traps. We noticed that when servicing the Scentry trap (with a screw lid) it proved easier to detach in comparison with the Trécé trap and the cup trap, which both had lids that popped off but would often become stuck together. Overall, the cup trap/Suzukii trap and the Scentry trap/Suzukii trap performed poorly, indicating that Suzukii trap is ineffective in our system irrespective of the trap used.

In the 2016 blueberry planting in Citra, significant differences were found in the numbers of male and female flies captured. The highest number of male flies was captured in the Scentry trap with Scentry lure; however, when the Trécé trap was equipped with Scentry lure and the Trécé trap with Trécé lure + yeast booster, there were no significant differences in male captures, indicating the efficiency of Scentry lure. It is apparent that Scentry lure out-competes the volatiles emitted in blueberries, because the trap-and-lure system continued to perform well during the fourth and fifth trapping periods when the blueberries were in their peak ripening period. Similar to the 2015 fruiting season, the cup trap with yeast bait captured a high percentage of females, but this was not different to the Scentry with Scentry lure, the Trécé trap with Trécé lure + yeast bait, and the Trécé trap with Scentry lure.

The overall trap captures for 2016 were lower than 2015. This may be due to the fluctuation in weather patterns among the two growing seasons. The mean temperature in Citra, Florida in 2015 was 23 °C, while the mean temperature in 2016 was 26 °C [24]. The optimal temperature for the development of the egg-to-adult stage of *D. suzukii* has been found to be 28.2 °C [6].

The lowest number of male flies was captured in the cup trap with Suzukii trap; however, this was not significantly different to the cup trap with Scentry lure and the Scentry trap with Suzukii trap. Similarly, the cup trap with Suzukii trap caught the lowest number of females numerically but was not significantly different to the Scentry trap with Suzukii trap and the cup trap with Scentry lure. These findings indicate the poor performance of Suzukii trap bait for both sexes. Another disadvantage in using this product is that the 200 mL of Suzukii trap often evaporated almost completely by the seventh day leaving behind a brown, thick, syrup-like residue. To solve this issue, we added a small amount of water to dilute the residue to release and record the trapped flies.

## 5. Conclusions

*Drosophila suzukii* is a relatively new and serious pest of berry plantings in Florida. Several traps, lures, and their combinations were evaluated over two years in three planting systems. A Scentry trap and Scentry lure provided the most consistent captures of *D*. *suzukii* throughout the entire season, attracting both male and female *D. suzukii*. Other traps, including a Trécé trap and Trécé lure + yeast bait, performed well but could be difficult and time consuming to deploy in the field. The effective performance of the Scentry trap and Scentry lure system could be due to the physical characteristics of the trap, including color, selectivity, and the attractiveness of the lure.

## Figures and Tables

**Table 1:** List of various treatments for three experiments conducted in Citra and Tallahassee (blueberry planting) and Citra (blackberry planting).

Treatments	Experiment 1 (Citra, FL, USA)	Experiment 2 (Tallahassee, FL, USA)	Experiment 3 (Citra, FL, USA)
I.	Cup trap + yeast bait *	Scentry trap + Scentry lure + 2% DS **	Cup trap + yeast bait *
II.	Cup trap + Scentry lure + 2% DS **	Scentry trap + yeast bait *	Cup trap + Suzukii trap
III.	Cup trap + Suzukii trap	Scentry trap + Trécé lure + 2% DS **	Trécé trap + Suzukii trap
IV.	Scentry trap + yeast bait + 2% DS **	Scentry trap + Suzukii trap	Trécé trap + Trécé lure + 2% DS **
V.	Scentry trap + Scentry lure + 2% DS **	Scentry trap + Trécé lure + 2% DS **	Scentry trap + Scentry lure + 2% DS **
VI.	Scentry trap + Suzukii trap	Trécé trap + Suzukii trap	Scentry trap + Suzukii trap
VII.	Trécé trap + yeast bait	Trécé trap + Trécé lure + yeast bait	--
VIII.	Trécé trap + Scentry lure + 2% DS **	--	--
IX.	Trécé trap + Suzukii trap	--	--

* Reference treatment. DS ** (detergent solution, 2% odorless detergent (Publix Super Market, Gainesville) and 98% water). Yeast bait had 29.6 g of yeast (Fleischmann’s Rapid Rise, ACH Food Companies, Inc. Cordova, TN, USA), 39.4 g of sugar (white granulated, Publix, Lakeland FL), and 590 mL of water (tap). Scentry lure had 41.5 g of lure (gel sachet), and Trécé lure had 37.9 g of lure mixture.

**Table 2:** *Drosophila suzukii* adults captured in the rabbiteye blueberry planting from 26 May 2015 to 30 June 2015 in Citra, Florida.

Treatments	No. of Flies Captured (Mean ± SE)	
	Male	Female
Trécé trap + Suzukii trap	5.6 ± 2.1dc	14.0 ± 4.5bc
Cup trap + Yt bait *	2.9 ± 1.2dc	7.7 ± 2.0bc
Trécé trap + T lure + Yt bait	13.9 ± 2.9ab	25.2 ± 3.1a
Scentry trap + Sc lure + 2% DS **	16.5 ± 6.3a	15.8 ± 3.5ab
Trécé trap + Sc lure + 2% DS **	4.7 ± 1.3dc	6.9 ± 1.6c
Cup trap + Sc lure + 2% DS **	3.7 ± 1.0dc	6.3 ± 1.0bc
Cup trap + Suzukii trap	1.0 ± 0.3d	5.8 ± 1.2c
Scentry trap + Suzukii trap	2.4 ± 0.8dc	6.6 ± 1.4bc
Scentry trap + Yt bait	8.4 ± 3.4bc	15.0 ± 3b

* Reference treatment. ** DS (2% detergent solution). Mean (± SE) number of adult flies within columns with different letters are significantly different (*p* ≤ 0.05; Tukey’s HSD test) from each other. Yt = Yeast bait, T lure = Trécé lure, and Sc lure = Scentry lure.

**Table 3:** Mean number of male and female adults captured in a rabbiteye blueberry planting from 19 May 2016 to 23 June 2016 in Citra, Florida.

Treatment	No. of Flies Captured (Mean ± SE)	
	Male	Female
Trécé trap + Suzukii trap	2.9 ± 1.0bc	3.60 ± 1.4ab
Cup trap + Yt bait *	2.30 ± 0.6bc	3.60 ± 0.8a
Trécé trap + Tr lure + Yt bait	3.6 ± 1.0abc	2.8 ± 0.7abc
Scentry trap + Sc lure + 2% DS **	7.3 ± 1.7a	3.0 ± 0.7ab
Trécé trap + Sc lure + 2% DS **	5.60 ± 1.4abc	3.50 ± 0.8ab
Cup trap + Sc lure + 2% DS **	2.4 ± 1.0cd	1.4 ± 0.5bcd
Cup trap + Suzukii trap	0.30 ± 0.2d	0.3 ± 0.1d
Scentry trap + Suzukii trap	0.40 ± 0.2d	1.0 ± 0.4dc
Scentry trap + Yt bait	4.50 ± 0.9abc	4.6 ± 0.9a

* Reference treatment. ** DS (2% detergent solution). Mean (± SE) number of adult flies within columns with different letters are significantly different (*p* ≤ 0.05; Tukey’s HSD test) from each other. Yt = Yeast bait, T lure = Trécé lure, and Sc lure = Scentry lure.

**Table 4:** Male and female *Drosophila suzukii* captured in blueberry planting from 18 April 2016 to 23 May 2016 in Tallahassee, Florida.

Treatments	Mean ± SE per Trap	
	Male	Female
Scentry trap + Suzukii trap	0.7 ± 0.4b	0.7 ± 0.3bc
Scentry trap + Yt bait *	0.7 ± 0.4b	0.9 ± 0.3bc
Scentry trap + Sc lure + 2% DS **	1.6 ± 0.4a	3.0 ± 0.6a
Scentry trap + Tr lure + 2% DS **	0.3 ± 0.1b	0.2 ± 1.0c
Sc trap + T lure + 2% DS **	0.1 ± 0.5b	0.5 ± 0.2bc
Trécé trap + Suzukii trap	0.5 ± 0.2b	1.0 ± 0.2bc
Trécé trap + T lure + Yt bait	0.2 ± 0.1b	0.10 ± 0.1c

* Reference treatment. ** DS (2% detergent solution). Mean (± SE) number of adult flies within columns with different letters are significantly different (*p* ≤ 0.05; Tukey’s HSD test) from each other. Yt = Yeast bait, T lure = Trécé lure, and Sc lure = Scentry lure.

**Table 5:** Male and female adults captured in blackberry planting from 4 June 2015 to 2 July 2015 in Citra, Florida.

Treatments	Mean ± SE per Trap	
	Male	Female
Cup trap + Yt bait *	0.2 ± 0.1b	0.8 ± 0.3ab
Trécé trap + Suzukii trap	0.1 ± 0.1b	1.3 ± 0.5ab
Trécé trap + T lure + 2% DS **	0.3 ± 0.2ab	1.4 ± 0.5ab
Scentry trap + Sc bait	2.0 ± 0.3a	2.3 ± 0.8a
Cup trap + Suzukii trap	0.1 ± 0.1b	0.7 ± 0.3b
Scentry trap + Suzukii trap	0.6 ± 0.3ab	0.7 ± 0.2ab

* Reference treatment. ** DS (2% detergent solution). Mean (± SE) number of adult flies within columns with different letters are significantly different (*p* ≤ 0.05; Tukey’s HSD test) from each other. Yt = Yeast bait, T lure = Trécé lure, and Sc lure = Scentry lure.
